# 

**Published:** 1909-09-11

**Authors:** 


### BOOKS RECEIVED.

James Maclehose and Sons.

"Clinical Manual for the Study of Diseases of the Throat." By James Walker Downie, M.B., F.F.P.S.G. 10s. net.

John Bale, Sons and Danielsson, Ltd.

"Blackwater Fever." By A. G. Newell, M.D., C.M., L.M., D.P.H. 5s. net.

Kegan Paul, Trench, Trübner and Co.

"The Book of the Golden Key." By Hugo Ames and Flora Hayter.

### APPOINTMENTS.

The Dundee Parish Council has appointed Dr. Eric A. Thomson, of Arbrough, medical officer of health.

Dr. C. M. L. Cowper has been appointed certifying surgeon under the Factory and Workshop Act for the Arnesby district of the county of Leicester.

Dr. Bertram George Belas, of Dublin, has been appointed second assistant resident medical officer by the Portsmouth Board of Guardians.

The Staffordshire Education Committee has appointed Miss Alice W. Maclean, M.B., Ch.B.Glasg., additional assistant medical inspector. There were thirty-three applicants for the post.

Dr. Schofield, medical officer to the Ravensthorpe district of the Dewsbury Union, has been appointed school medical officer of Preston, and Dr. Pearson, of Dublin, has been appointed to succeed him at Ravensthorpe.

It is officially announced that Dr. Thomas H. Bryce, lecturer on anatomy at Queen Margaret College, has been appointed to the Chair of Anatomy at the University of Glasgow in succession to Professor Cleland. The new professor is M.A., M.D. (gold medallist), of Edinburgh University, and a Fellow of the Scottish Royal and Antiquarian Societies. He is the author of the section Embryology in the eleventh edition of Quain, and has written many treatises and papers on anatomical subjects.

The following gentlemen have been selected as house officers at St. Thomas' Hospital from Tuesday, September 7, 1909 : Casualty officers and resident anæsthetists : W. B. Johnson, M. L. C. Irvine, E. W. Witney, C. T. V. Benson, C. D. H. Corbett, and B. A. Cheadle. Casualty assistants : H. Bowring and C. Gouldesbrough. Resident house physicians (from August 24, 1909) : M. W. Baker, L. B. Perry, L. S. T. Burrell, H. B. Wilson, and G. E. Thornton. Resident house surgeons (from August 24, 1909) : J. B. Mennell, G. B. Wainwright, W. Harmens, and R. Cox. House surgeon to block 8 : W. R. Bristow. Obstetric house physicians : (*Senior*) P. T. Harper, and (*Junior*) E. L. Fyffe. Ophthalmic house surgeon : E. M. Parsons-Smith. Clinical assistants : (*Senior*) W. Ibbotson, B. C. Maybury, J. C. Marklove, J. S. Hopwood, W. Ibbotson, F. J. Aldridge, J. L. Graham-Jones, and F. C. Pridham.

### THE GRADUATES' MEDICAL SCHOOLS.

DIARY FOR THE WEEK, SEPTEMBER 13 TO 18.

**MEDICAL GRADUATES' COLLEGE AND POLYCLINIC, 22 Chenies Street, W.C.**

**Sept. 13**, College re-opens.

**At 4 p.m.**

**Sept. 14**, Dr. R. Hutchison, **Medical.**

**Sept. 15**, Mr. Arthur Edmunds, **Surgical.**

**Sept. 16**, Sir Jonathan Hutchinson, **Surgical.**

**Sept. 17**, Dr. William Hill, **Ear, Nose, and Throat.**

Dr. Waugh, district medical officer for the Skipton sub-district, has resigned. Dr. Waugh is leaving the district, and has also resigned his appointment as medical officer to the Skipton and District Infectious Diseases Hospital.

Dr. Littlejohn, medical superintendent at the Hanwell Schools of the Central London School District, has resigned. He has held the position since 1870. The managers have resolved to accept the resignation with regret, and to send a letter of sympathy to Dr. Littlejohn, whose retirement is due to a breakdown.

Four children died recently at Rotterdam. Their deaths, at first supposed to be caused by the eating of poisoned sweets, are now proved to have resulted from Asiatic cholera. The body of a sea captain whose death had given rise to suspicion has been delayed burial in order that an examination may be made. About twenty-five persons, of whom fourteen are children, are under observation. Stringent measures have been taken by the authorities to prevent the spread of the disease.

The Sevenoaks District Council having recently appointed an official of the Royal Society for the Prevention of Cruelty to Animals to act as "honorary sanitary inspector," the National Federation of Meat Traders, acting at the instance of the butchers of the locality, took counsel's opinion on the appointment. This opinion is to the effect that the appointment is illegal, and in these circumstances the federation has called upon all meat traders in Sevenoaks to refuse admission to their premises to the honorary sanitary inspector. At the same time the local butchers are advised to allow members of the local authority to visit and inspect their premises. Pending the return of the Sevenoaks District Council, which is now on vacation, the honorary sanitary inspector has been written to and requested not to make any visits until the council meets and considers the protest of the federation.

